# Parental care and depressive symptoms among Chinese medical students: roles of empathy and gender

**DOI:** 10.1186/s12909-022-03524-2

**Published:** 2022-06-11

**Authors:** Yiran Geng, Wenjing Fei, Zhengyu Tang, Shaishai Wang, Jiachun Yu, Ming Zhang, Tianyang Zhang

**Affiliations:** 1 School of Public Health, Medical College of Soochow University, Suzhou, China; 2 Department of Psychology, Suzhou University of Science and Technology, Suzhou, China

**Keywords:** Subclinical depressive symptoms, Parental care, Empathy, Medical students, Mental health

## Abstract

**Background:**

Medical students in China who face the dual pressure of study and employment tend to experience subclinical depressive symptoms. Parental care plays an important direct and indirect role in the psychological development of medical students, and the extent and mechanism of this role urgently need to be studied and discussed.

**Methods:**

After simple random sampling and screening of valid questionnaires, data from a total of 924 people were used. The participants completed the parental bonding instrument, self-rating depression scale, Chinese version of the Jefferson empathy scale-medical student edition and self-rating anxiety scale to evaluate parental care, empathy, depressive symptoms and anxiety. The data were statistically processed using a descriptive analysis, correlation analysis and test of moderated mediation.

**Results:**

Maternal care had a significant negative predictive effect on depressive symptoms among medical students. Strong maternal care can reduce the occurrence of depressive symptoms in medical students. Empathy played a positive mediating role such that both types of empathy could alleviate the effect of weak maternal care on the depressive symptoms of medical students. However, neither cognitive empathy nor affective empathy played a mediating role in the relationship between paternal care and depressive symptoms. Nevertheless, the relationship between maternal care and empathy was moderated by the medical students’ gender.

**Conclusions:**

The effect of this relationship on female medical students deserves special attention. The results of this study provide a reference and basis for the adjustment of medical education. This study could also help in the design of effective psychological intervention measures to reduce the degree of depressive symptoms and enhance personal empathy.

## Background

The incidence of depressive symptoms is increasing annually [1–3]. According to the latest statistics from the World Health Organization (WHO), the prevalence rate of depression in China is 6.9% [1], indicating that China has one of the highest burdens of depressive symptoms worldwide [3].

In a systematic review of depressive symptoms among medical students, the overall prevalence of depressive symptoms among medical students was reported to be higher than that in the general population, with conflicting findings regarding whether student depression varies by undergraduate grade, gender, or other characteristics [4].

### Subclinical depressive symptoms

Subclinical depressive symptoms are risk factors for clinical depression [5, 6], which constitutes a depressive state that does not meet the DSM-IV criteria for the symptoms or course of major depression [1, 7]. Subclinical depression has a high incidence in the general population, ranging from 1.4 to 17.2% in the community. Among medical students in China, the incidence of subclinical depression is as high as 32% [2]. Social dysfunction is present not only in patients with clinical depression (MDD) but also in patients with subclinical depressive symptoms [5, 7]. Some studies have shown that abnormal empathy in patients with subclinical depression may contribute to the development of MDD [1, 5, 6].

Many factors cause subclinical depression in medical students. First, in the current educational framework in China, Chinese medical students undergo five years of undergraduate study, with the first and second years focusing on the basics. Following the third year, medical students are exposed to more specialized knowledge and laboratory operations. Then, fourth- and fifth-grade students are assigned to corresponding hospitals for clinical practice [8]. Five years of intense medical education can easily lead to psychological imbalances among medical students [8, 9]. If these factors cannot be effectively alleviated for a long period, they aggravate negative emotions, such as subclinical depressive symptoms, which is consistent with the results of previous studies [10, 11].

### Effects of parenting care on subclinical depressive symptoms and depression

Parental rearing style is closely related to the physical and mental health development of medical students [12]. The term “parental rearing styles” was first formulated in 1959 by Schaefer, and these styles have a significant impact on children’s psychological formation [12, 13]. There are four parental styles: authoritative (high warmth and low control), overprotective (high warmth and high control), authoritarian (affective coldness and high control) and neglecting (affective coldness and low control). In 1979, Parker divided this concept into three major factors: maternal or paternal caring, maternal or paternal encouraging autonomy and maternal or paternal controlling [14]. It is worth noting that the influence of parenting style on children is generally formed before the age of 16, with little change thereafter [15, 16].

A regression analysis [17] of subclinical depressive symptoms by parenting style found that increased parental care is associated with a lower risk of subclinical depressive symptoms among children and adolescents. Studies have shown that parental care is more closely associated with depressive symptoms than parental encouragement or control [17, 18]. Improving parental care may contribute to the treatment and prevention of adolescent subclinical depressive symptoms [19]. Parental care has an impact on children’s mental health in childhood [20–22]. Medical college students, who are obviously in the stage of adulthood, are in a contradictory stage of independence and dependence in life. Moreover, the social value of medical professionals can be reflected only through long-term experience accumulation; thus, this contradiction may be intensified [23]. Notably, parental care not only affects medical students’ emotions and produces depressive symptoms but also affects their empathy [19, 24–29].

### The mediating effect of empathy

Empathy is a multidimensional concept. Currently, empathy is understood as both emotional and cognitive [6, 30]. Affective empathy is an individual’s emotional response to the emotional states of others. Cognitive empathy [31] is an individual’s ability to understand the thoughts and feelings of others without being affected by emotions. Therefore, the empathic two-system hypothesis and empathic two-process model were formed [5, 32, 33]. Empathy is an important part of doctor–patient relationships and an important personality characteristic of Chinese medical students. However, studies have shown that the level of empathy declines during undergraduate medical education [25, 34].

Medical students with empathy are more likely to have subclinical depressive symptoms than nonmedical students [6, 35], likely because the academic pressure on medical students is high and subsequent clinical practice has a negative impact on communication with patients [36]. Some scholars believe that high levels of emotional empathy among medical students are positively correlated with subclinical depressive symptoms [37, 38]. In contrast, cognitive empathy was found to be irrelevant to or negatively associated with subclinical depressive symptoms. Therefore, based on these theoretical and empirical findings, we hypothesize that empathy is a mediator of the relationship between parental care and subclinical depressive symptoms [6]. A correlation exists between empathy and parental care among college students. For example, studies [19, 24] have shown that excessive parental care increases cognitive empathy and decreases affective empathy, resulting in psychological stress in college students.

### Gender as a moderator

This association is different for populations with different characteristics, such as gender, age, living environment, and educational background [20, 21]. A previous study [20] found that boys are more likely than girls to experience harsh punishment, excessive interference and denial from their fathers, while girls are more likely to receive emotional warmth and understanding from their mothers. This finding can likely be attributed to traditional Chinese culture, in which society and families have more expectations and requirements for men [21, 39, 40].

Some studies [1, 6] have observed gender differences in empathy, with women generally showing more compassionate care. There are also gender differences in subclinical depressive symptoms. Women with higher compassionate care are more likely to develop subclinical depressive symptoms [23]. A previous study [41] of the relationship between maternal parenting style and the empathic ability of Chinese medical students showed that in terms of gender, female medical students scored significantly higher than male medical students in empathic ability, and there were differences in the scores between male and female medical students in the dimensions of their maternal parenting style. It is reasonable to hypothesize that gender may play a moderating role in the relationship among parental care, empathy and subclinical depressive symptoms.

### The present study

Previous studies have focused on the relationship between parental overcontrol and depressive symptoms in medical students [6, 16, 17, 19]. However, few studies have focused on the relationship among parental care, empathy and subclinical depressive symptoms in medical students of different genders, and few detailed studies have investigated whether empathy plays a mediating role [6, 39, 40, 42]. Detailed and comprehensive studies explaining the mechanism that mediates this association and the moderating factors that affect this relationship are lacking.

We therefore propose the following three hypotheses.

Hypothesis 1: Parental care has a reverse predictive effect on depressive symptoms.

Hypothesis 2: The effect of parental care on depressive symptoms is mediated by empathy.

Hypothesis 3: Gender moderates the relationship among the above three factors (two pathways before and after the model).

## Methods

### Participants

In this cross-sectional study, we recruited 945 medical students at the Medical College of Soochow University from grade 1 to grade 5 by stratified random sampling in November 2020. Exclusion criteria included self-reported presence of a medical or psychiatric disorder diagnosed by psychiatrists, and long-term use or dependence on psychotropic prescription drugs. After removing the invalid data, a total of 924 sample data points were collected, with a good recovery rate of 98%. The participants provided informed consent before the survey and were informed of their right to withdraw from the survey. Students who wanted to know the results of their own questionnaire were provided with basic survey information after participating in the survey. The project was approved by the Ethics Committee of Soochow University (ECSU-2019000154).

### Procedure

In this study, the scope of the investigation was undergraduates of the Medical College at Soochow University, who are divided into 5 grades (grades 1 to 5). Students in the first and fifth grades accounted for 18.13, 23.35, 22.94, 19.35 and 16.23% of the sample, respectively. Reviewing the literature and referring to previous survey results, the current incidence of depressive symptoms among medical students in China is approximately 32% [2]. We set the tolerances at δ = 0.032, π = 0.32, α = 0.05, and u _α/2_ = 1.96. The sample size estimation formula was $n=\frac{u_{a/2}^2\pi \left(1-\pi \right)}{\delta^2}$, and the required sample size was 816 people. Considering the survey recovery rate and efficiency, the sample size was set to 940 people. Simple random sampling was implemented in each grade. Trained psychological test administrators organized students to complete the questionnaire anonymously. To ensure the validity of the data, we deleted participants who gave consistent answers to all items in the questionnaire.

### Measures

#### Parental Bonding Instrument (PBI)

Parental rearing styles are relatively stable behaviour styles of parents in the activities of raising and educating their children. The PBI is a summary of the characteristics of parents’ various rearing behaviours. To define and evaluate the effective composition of parental parenting behaviours, Parker compiled the PBI questionnaire in 1979 [13] based on attachment theory. Numerous studies have shown that the PBI has good reliability and no gender differences, and the scores are less affected by the subjects’ levels of depression and major life events than other instruments. In China, Yang et al. examined the applicability of the PBI to Chinese college students and revised it accordingly. Three factors, care, encouragement and control, were extracted through exploratory factor analysis [41]. Using a 4-point scale, “0″ represents “strongly disagrees”, “1″ represents “relatively disagrees”, “2″ represents “fairly agree”, and “3″ represents “strongly agree”. The Cronbach’s α of the maternal version (PBI-M) and the paternal version (PBI-F) in this study were 0.821 and 0.733, respectively. Therefore, we used this scale to measure the impact of perceived parenting style during childhood on medical students.

#### Self-Rating Depression Scale (SDS)

The SDS is a tool used to measure depression. The SDS was developed in 1965–1966 by William W.K. Zung [43], a professor at Duke University. The SDS has been introduced domestically since the early 1980s. Test application indicated that the SDS was also suitable for the measurement of depressive symptoms in China and developed the norm of the SDS [42]. The SDS includes 20 items, and each item is composed of four grades. Two items are related to psychosocial and affective symptoms, eight items are related to somatic disorders, two items are related to psychomotor disorders, and eight items are related to depressive psychological disorders. Each entry is graded on a 4-point scale. The participants selected 1 (never once in a while), 2 (sometimes), 3 (often), or 4 (always) according to the time frequency that best suited their situation. Of the 20 items, 10 are positively worded and reverse scored, and the remaining 10 are negatively worded. The items were scored 1–4 [42]. The scale is easy to use and can intuitively reflect the subjective feelings of the subjects. The Cronbach’s α was 0.752**.**

#### Chinese version of the Jefferson Empathy Scale Medical Student Edition (JSPE-S)

This scale adopts a 7-level score with 10 positively scored items, such as “understanding patients’ body language in the doctor–patient relationship is as important as verbal communication”, and 10 negatively scored items, such as “attention to the patient’s personal experience will not have an impact on the treatment outcome”. There are three factors: perspective taking, compassionate care, and standing in the patient’s shoes [25]. The overall score ranges from 20 to 140, with higher scores associated with higher levels of empathy. Based on the characteristics of the items and related literature, this study incorporated standing in the patient’s shoes into perspective taking to represent the cognitive empathy score [10, 26] and compassionate care to represent the affective empathy score. The Cronbach’s α was 0.731**.**

#### Self-Rating Anxiety Scale (SAS)

The Self-Rating Anxiety Scale was developed by W.K. Chung in 1971 [27]. The SAS is recommended for use in adults with anxiety symptoms and has a wide range of applications similar to the SDS, which is also suitable in China [28]. The scale is used to assess patients’ subjective feelings of anxiety and their changes during treatment. The scale consists of 20 questions, and items 5, 9, 13, 17 and 19 are reverse scored. A 4-point scale was used to assess the frequency of symptoms described by an item as follows: 1-no or little time; 2-a small amount of time; 3-a considerable amount of time; and 4-most or all the time. The Cronbach’s α was 0.754**.**

#### Statistical analyses

SAS version 9.4 software was used to conduct a descriptive analysis of each variable by t test (after verification of the normality of the distribution) and analysis of variance, followed by a linear correlation analysis of the correlation between the variables. Then, the PROCESS macro in SPSS version 21.0 was used to analyse the mediating effect of empathy and the moderating effect of gender. Model 7 [44] (a mediated moderation model and moderator moderating the first half of the pathway) was selected in the SPSS macro program to analyse the mediating effect and the moderated mediating model, and standardized regression coefficients were obtained. A *p* < 0.05 was considered statistically significant.

## Results

### Participant characteristics

Among the participants, 289 were male and 635 were female, accounting for 31.3 and 68.7% of the sample, respectively. Of these participants, 597 had no siblings (64.6%), 859 (93.0%) had friends, and 632 (68.4%) liked sports. The number of freshman to fifth-year students was 159 (17.2%), 225 (24.4%), 212 (22.9%), 188 (20.3%), and 140 (15.2%), respectively. The average usage time of new media every day was 1–3 h (12.6%), 4–5 h (30.4%), 6–7 h (24.7%), and over 8 h (32.3%), respectively (Table 1).

**Table 1:** Characteristics of study variables among medical students by demographic factors (M ± SD) (*n =* 924)

	*n*	Perspective taking	Compassionate care	Depressive symptoms	Anxiety	Maternal care	Maternal encourage	Maternal control	Paternal care	Paternal encourage	Paternal control
**Gender**		0.57	<0.001	0.07	0.99	0.99	<0.01	0.06	0.69	0.94	0.46
Male	289	66.92 ± 9.05	38.12 ± 7.08	34.72 ± 8.39	32.19 ± 8.39	24.62 ± 5.13	12.09 ± 3.80	6.34 ± 2.93	20.51 ± 6.25	11.36 ± 3.54	5.20 ± 2.82
Female	635	67.26 ± 8.00	39.67 ± 5.89	35.79 ± 8.12	32.20 ± 7.56	24.66 ± 5.08	12.84 ± 3.53	5.97 ± 2.68	20.69 ± 6.43	11.34 ± 3.59	5.35 ± 2.70
**Onlychild**		0.95	0.19	0.38	0.15	<0.001	0.28	<0.01	<0.001	<0.001	<0.05
Yes	597	67.17 ± 8.59	38.98 ± 6.55	35.28 ± 8.35	31.93 ± 7.79	25.09 ± 5.04	12.70 ± 3.69	6.30 ± 2.90	21.25 ± 5.74	11.67 ± 3.20	5.45 ± 2.71
No	327	67.13 ± 7.88	9.55 ± 5.88	35.78 ± 7.97	32.69 ± 7.89	23.84 ± 5.09	12.43 ± 3.52	5.70 ± 2.47	19.53 ± 7.27	10.75 ± 4.11	5.03 ± 2.76
**Friend**		<0.05	0.50	<0.001	<0.01	<0.001	<0.01	0.18	0.11	0.3	0.83
Yes	859	67.31 ± 8.26	39.22 ± 6.40	35.17 ± 8.09	32.02 ± 7.75	24.80 ± 4.99	12.69 ± 3.60	6.05 ± 2.75	20.73 ± 6.35	11.38 ± 3.57	5.31 ± 2.75
No	65	65.02 ± 9.16	38.68 ± 5.08	9.19 ± 9.07	34.55 ± 8.50	22.68 ± 5.96	11.52 ± 3.86	6.52 ± 2.94	19.42 ± 6.64	10.91 ± 3.66	5.23 ± 2.61
**Sport**		<0.001	0.33	<0.001	0.09	0.14	0.95	0.29	0.33	0.90	0.48
Yes	632	67.88 ± 8.13	39.32 ± 6.35	34.58 ± 7.94	31.90 ± 7.69	24.82 ± 4.89	12.60 ± 3.60	6.15 ± 2.77	20.78 ± 6.41	11.36 ± 3.60	5.35 ± 2.81
No	292	65.58 ± 8.54	38.89 ± 6.26	37.36 ± 8.50	32.84 ± 8.09	24.28 ± 5.49	12.62 ± 3.70	5.94 ± 2.75	20.34 ± 6.29	11.33 ± 3.54	5.21 ± 2.58
**Grade**		<0.01	<0.001	<0.01	<0.05	<0.05	0.16	0.62	0.17	0.79	0.12
2020	159	69.11 ± 7.99	39.15 ± 6.39	33.34 ± 7.73	30.58 ± 6.89	25.69 ± 4.52	12.96 ± 3.47	5.98 ± 2.64	21.43 ± 6.17	11.57 ± 3.50	5.28 ± 2.77
2019	225	67.04 ± 8.21	38.17 ± 6.67	36.32 ± 8.61	33.05 ± 8.69	24.72 ± 5.11	12.98 ± 3.40	6.00 ± 2.80	20.72 ± 6.56	11.44 ± 3.57	5.32 ± 2.73
2018	212	67.49 ± 9.23	40.35 ± 5.99	35.63 ± 7.75	32.16 ± 7.93	24.54 ± 5.29	12.31 ± 3.80	6.14 ± 2.92	20.18 ± 6.82	11.15 ± 3.85	5.00 ± 2.68
2017	188	66.51 ± 7.83	39.95 ± 5.63	36.02 ± 8.37	32.14 ± 7.42	23.80 ± 5.40	12.36 ± 3.81	5.97 ± 2.59	19.98 ± 6.29	11.22 ± 3.52	5.27 ± 2.70
2016	140	65.46 ± 7.80	38.04 ± 6.63	35.46 ± 8.28	32.79 ± 7.56	24.66 ± 4.79	12.40 ± 3.62	6.40 ± 2.87	21.16 ± 5.60	11.43 ± 3.33	5.81 ± 2.80
**E-time**		<0.05	0.17	<0.001	<0.001	0.55	0.61	0.28	0.53	0.62	0.34
1 h ≤ Time ≤ 3 h	116	69.04 ± 8.59	40.21 ± 6.67	33.41 ± 8.66	30.93 ± 7.33	25.17 ± 5.34	12.67 ± 3.52	6.13 ± 2.73	20.54 ± 6.83	11.46 ± 3.81	5.35 ± 3.04
4 h ≤ Time ≤ 5 h	281	67.60 ± 7.99	38.96 ± 6.24	34.47 ± 7.39	31.03 ± 7.03	24.77 ± 4.85	12.82 ± 3.58	5.86 ± 2.64	21.03 ± 6.17	11.51 ± 3.46	5.02 ± 2.50
6 h ≤ Time ≤ 7 h	228	67.12 ± 7.58	39.42 ± 6.42	35.58 ± 8.02	32.50 ± 7.82	24.65 ± 4.91	12.63 ± 3.36	5.99 ± 2.76	20.54 ± 6.39	11.18 ± 3.65	5.41 ± 2.97
Time ≥ 8 h	299	66.10 ± 9.04	38.92 ± 6.06	37.04 ± 8.68	33.49 ± 8.54	24.38 ± 5.38	12.35 ± 3.92	6.35 ± 2.89	20.45 ± 6.34	11.32 ± 3.53	5.46 ± 2.62

E-time = new media screen time, M ± SD = Mean score ± standard deviation

### Descriptive analysis

After the analysis of sociodemographic and other variables, significant differences were found in the scores of compassionate care by gender (*t* = 3.47, *df* = 922, *p* < .001), with females reporting (39.67 ± 5.89) higher scores than males (38.12 ± 7.08). There was no significant difference in perspective taking, depressive symptoms and anxiety by gender. Being an only child had a significant effect on the parental rearing style. Medical students without siblings tended to experience higher parental care (21.25 ± 5.74) (*t* = 3.95, *df* = 922, *p* < .001), maternal control (6.30 ± 2.90) (*t* = 3.15, *df* = 922, *p* < .01) and paternal encouragement (11.67 ± 3.20) (*t* = 3,77, *df* = 922, *p* < .001) than students with siblings. The empathy, depression and anxiety scores were not significantly different in students with or without siblings.

Medical students who had friends had higher scores for perspective taking (67.31 ± 8.26) (*t* = 2.33, *df* = 922, *p* < .05) and lower levels of depressive symptoms (35.17 ± 8.09) (*t* = −3.82, *df* = 922, *p* < .001) and anxiety (32.02 ± 7.75) (*t* = −2.53, *df* = 922, *p* < .01). Students who liked sports had higher perspective taking scores (67.88 ± 8.13) (*t* = 3.54, *df* = 556, *p* < .001) and lower depressive symptoms (34.58 ± 7.94) (*t* = −4.73, *df* = 533, *p* < .001). With the increase in grade, the two types of empathy, depressive symptoms, and anxiety among the medical students changed to various degrees. With regard to scores, perspective taking (*F* = 3.66, *df* = 4, *p* < .01) and compassionate care (*F* = 5.20, *df* = 4, *p* < .001) decreased, while the degree of depressive symptoms (*F* = 3.53, *df* = 4, p < .01) and anxiety increased (*F* = 2.57, *df* = 4, *p* < .05). With the increase in the time spent using new media, the scores of the medical students in perspective taking (*F* = 3.85, *df* = 3, *p* < .05) continuously decreased, whereas the degree of depressive symptoms (*F* = 7.41, *df* = 3, *p* < .001) and anxiety (*F* = 5.83, *df* = 3, *p* < .001) continued to rise. Parenting styles were not significantly different among students who exercised or did not and students with different durations of new media use. Details are shown in Table 1.

### Correlation analysis

As shown in Table 2, parental care was negatively correlated with depressive symptoms (*t* = −0.34, *p* < .001; *t* = −0.20, *p* < .001) but was positively correlated with perspective taking (*t* = 0.18, *p* < .001; *t* = 0.15, *p* < .001) and compassionate care (*t* = 0.10, *p* < .01; *t* = 0.10, *p* < .01). The two types of empathy were negatively correlated with depressive symptoms (*t* = −0.30, *p* < .001; *t* = −0.21, *p* < .001). Anxiety was positively correlated with depressive symptoms (*t* = 0.79, *p* < .001) and negatively correlated with parental care (*t* = −0.33, *p* < .001; *t* = −0.18, *p* < .001), perspective taking (*t* = −0.24, *p* < .001) and compassionate care (*t* = −0.23, *p* < .001).

**Table 2:** The linear correlations among the study variables (*n =* 924)

	Perspective taking	Compassionate care	Depressive symptoms	Anxiety	Maternal care	Paternal care
**Perspective taking**	1					
**Compassionate care**	0.46^***^	1				
**Depressive symptoms**	−0.30^***^	−0.21^***^	1			
**Anxiety**	−0.24^***^	−0.23^***^	0.79^***^	1		
**Maternal care**	0.18^***^	0.10^**^	−0.34^***^	−0.33^***^	1	
**Paternal care**	0.15^***^	0.10^**^	−0.20^***^	−0.18^***^	0.42^***^	1

^*^p < .05, ^**^p < .01, ^***^p < .001

### The mediating effect of empathy

First, we found that both types of empathy played a positive mediating role in the relationship between maternal care and depressive symptoms but did not play a mediating role in the relationship between paternal care and depressive symptoms. As shown in Table 3, maternal care had a direct and negative effect on depressive symptoms (*β* = −0.54, *t* = −10.88, *p* < .001). When the two types of empathy were included in the mediation model, the direct negative effect of maternal care on depressive symptoms decreased (*β* = −0.49, *t* = −4.87, *p* < .001). The effect of maternal care on perspective taking was *β* = 0.23, *t* = 5.10, *p* < .001, and the effect of maternal care on compassionate care was *β* = 0.12, *t* = 2.97, *p* < .001, both of which were positive. The negative effects of empathy on depressive symptoms were *β* = −0.19, *t* = −2.33, *p* < .05 and *β* = −0.10, *t* = −2.75, *p* < .01. Bootstrapping showed that empathy explained the relationship between maternal care and depressive symptoms by perspective taking (indirect effect = 0.06, 95% CI = 0.02–0.08) and compassionate care (indirect effect = 0.01, 95% CI = 0.001–0.03).

**Table 3:** The mediating role of empathy between maternal care and depressive symptoms (*n =* 924)

	Model 1		Model 2		Model 3		Model 4	
	(Depressive symptoms)		(Perspective taking)		(Compassionate care)		(Depressive symptoms)	
Variables	*β*	*t*	*β*	*t*	*β*	*t*	*β*	*t*
Maternal care	−0.54	−10.88^***^	0.23	5.10^***^	0.12	2.97^***^	−0.49	−4.87^***^
Perspective taking							−0.19	−2.33^*^
Compassionate care							−0.1	−2.75^**^
R^2^	0.11		0.17		0.1		0.42	
*F*	118.44^***^		25.98^***^		8.79^**^		49.98^***^	

^*^p < .05, ^**^p < .01, ^***^p < .001

### Moderation by gender

**Fig. 1:**
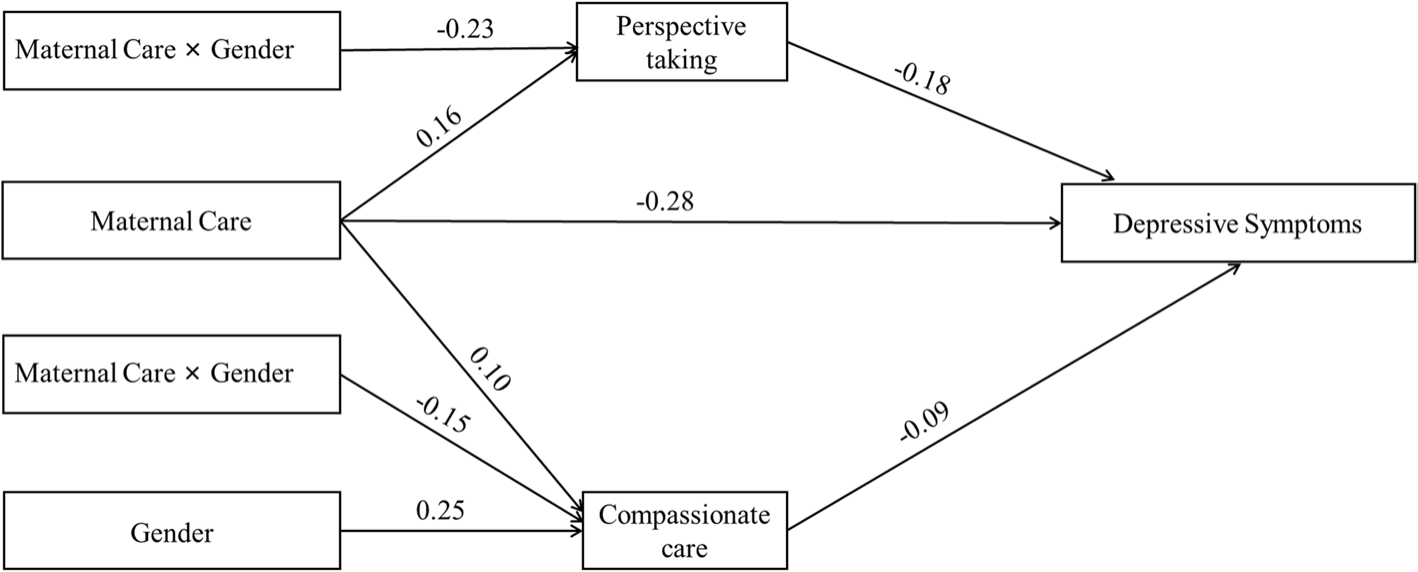
The moderated mediation model with maternal care. (*n =* 924)

**Fig. 2:**
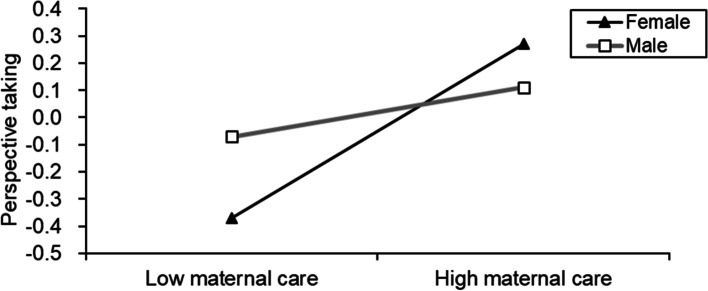
The simple slope diagram of the moderating effect of gender on maternal care and perspective taking. (*n =* 924)

**Fig. 3:**
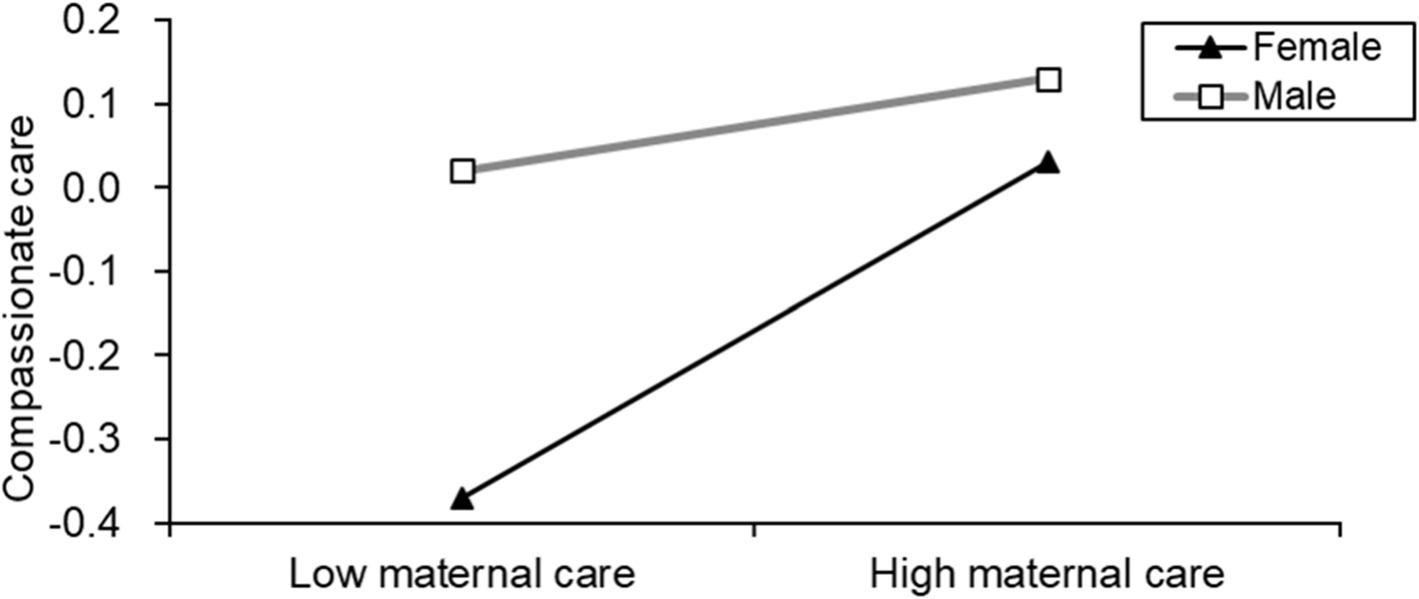
The simple slope diagram of the moderating effect of gender on maternal care and compassionate care. (*n =* 924)

When the gender variable was added to the model, its moderating effect had an impact on the path effect test of the first half of the model. The overall framework is shown in Fig. 1. Table 4 shows that maternal care and sex had significant predictive effects on perspective taking and compassionate care (*β* = −0.23, *t* = −3.38, *p* < .001; *β* = −0.15, *t* = −2.09, *p* < .05) but no significant predictive effect on depressive symptoms. Further simple slope analysis (Figs. 2 and 3) showed that female medical students had a higher slope in both perspective taking and compassionate care than in maternal care. The influence of maternal care on female medical students’ empathy was greater than that on male medical students’ empathy.

**Table 4:** The mediating effect of gender moderation on maternal care (*n =* 924)

	Model 1		Model 2		Model 3	
	(Perspective taking)		(Compassionate care)		(Depressive symptoms)	
Variables	*β*	*t*	*β*	*t*	*β*	*t*
Maternal care	0.16	4.96^***^	0.10	3.05^**^	−0.28	−9.30^***^
Gender	0.07	0.98	0.25	3.43^***^		
Maternal care× Gender	−0.23	−3.38^***^	−0.15	−2.09^*^		
Perspective taking					−0.18	−5.26^***^
Compassionate care					−0.09	−2.73^***^
*R*^*2*^	0.08		0.03		0.21	
*F*	7.45^**^		3.17^**^		24.00^**^	

^*^p < .05, ^**^p < .01, ^***^p < .001

## Discussion

The mental health and empathic ability of medical students play an important role in their academic lives and interpersonal communication [29]. Most medical students in this research showed some degree of depressive symptoms. Moreover, empathy plays an important role in the career development of medical workers and the doctor–patient relationship. Symptoms of subclinical depression increased, but not continuously; this finding requires further study [6, 10].

Subclinical depressive symptoms were negatively correlated with parental care, and low parental care received by medical students was more likely to produce depressive symptoms (consistent with hypothesis 1). In addition, the present study found a mediating role of the two types of empathy in the relationship between maternal care and subclinical depressive symptoms, which is consistent with hypothesis 2. However, a moderating role of gender was found only in the relationship between maternal care and empathy, which is not completely consistent with hypothesis 3.

### The mediating role of empathy

Both types of empathy played a partial mediating role in the relationship between maternal care and subclinical depressive symptoms, but perspective taking played a greater role (consistent with hypothesis 2). The results suggest that less maternal care is detrimental to the development of children’s empathy ability and thus increases the likelihood of subclinical depressive symptoms. One difference between this study and previous studies is that empathy did not mediate the relationship between paternal care and subclinical depressive symptoms. Therefore, the mediating factors of paternal care and depressive symptoms deserve further study. After considering the entire mediation model, we further analysed each chain.

Cognitive empathy has been shown to be negatively correlated with parental care in previous studies involving nonmedical students, while affective empathy has been shown to be positively correlated with parental care [8, 22, 45]. The first half of the mediation process in the present study showed that maternal care had the same effect on affective empathy and cognitive empathy in medical students. This finding highlights the particularity of medical students and the greater influence of maternal care on their empathic ability during college life and further shows that active maternal care plays an important role in the mental health development of medical students.

Specifically, maternal care has a greater influence on students primarily before the age of 16, and children’s interpersonal communication styles with others are mainly shaped by interactions with their parents at a young age. However, parents who exhibit negative parental care often ignore the thoughts or emotions of their children [46]. Medical students entering medical school are exposed to environmental influences that amplify this deficiency, which ultimately hinders the development of their ability to empathize [10, 47]. These findings call for the need to consider that maternal care plays a positive role in medical students’ empathy. Medical schools can examine factors that influence the degree of empathy among medical students and identify learning gaps [26] where interventions can reinforce students’ perceptions. Quantitative evaluations are an effective way, such as using the medical student edition of the Jefferson Empathy Scale [29].

In the second half of the mediation process, we found that perspective taking and compassionate care were both negatively associated with depressive symptoms. A review by Schreiter and colleagues shows that depression and depressive symptoms are strongly associated with affective and cognitive empathy [5]. This is likely due to the heavy academic burden of medical students in Chinese medical colleges and universities [8, 25], which has a great influence on their empathy development [29]. Furthermore, difficult doctor–patient relationships during an internship further deepen the negative development of medical students’ empathy and subclinical depressive symptoms [10]. This finding demonstrates that empathy, among other factors, may be an important cause of subclinical depressive symptoms [48]. In our research, medical students with low empathy (especially cognitive empathy) were our focus group and are prone to subclinical depressive symptoms both in medical school and in clinical practice. Clinical rotations can help students gain additional insights through interactions with patients and put themselves in the patient’s shoes during health care treatment [47], which can help medical students adapt to clinical work and reduce psychological resistance [49].

### The moderating effect of gender

In contrast to hypothesis 3, we found that gender moderated the indirect effect in the relationship between maternal care and subclinical depressive symptoms. The specific moderating effect occurred in the first half of the mediating chain, and the combined effect of gender and maternal care moderated not only perspective taking but also compassionate care. Specifically, maternal care had a greater impact on the two types of empathy among female medical students. These results are consistent with those of previous studies [49, 50].

A possible explanation is that female medical students are more likely to have more emotional output to maintain harmonious interpersonal relationships. In contrast, male medical students begin to yearn for an independent life. In addition, this finding is consistent with relevant research involving Chinese medical students [10], suggesting that female medical students are more likely to be sensitive to parental care, especially from their mothers, which may have a negative impact on their mental health. Furthermore, some studies [29, 49] have shown that due to academic particularity, female medical students are more likely to experience emotional fluctuations and both types of empathy in medical course learning than male medical students. Comparatively, male medical students are better than female students in mastering courses, especially in practical skills [51].

Our results also show that gender did not moderate the relationship between empathy and depressive symptoms and only moderated the relationship between maternal care and both types of empathy, which is not consistent with hypothesis 3. According to previous data screening, gender regulates the relationship between cognitive empathy and depressive symptoms [31]. Some studies have also shown that gender does not regulate the association between empathy (i.e., affective empathy and cognitive empathy) and depressive symptoms [52]. Few studies have provided sufficient evidence to prove that gender moderates this relationship [5]. This area requires further study to determine whether other objective factors alter the effect of this regulation.

### Limitations and implications

This study has some limitations and deficiencies. First, this study is a single-centre study. Multicentre studies should be conducted in the future to further verify and generalize the results [10]. Second, the study used subjective survey methods; it is therefore possible that answers may have been given based on social desirability. The Chinese versions of these four scales have been verified in previous studies [47]. However, language conversion problems due to translation may have affected the experiment to some extent. Third, our study found more evidence of the relationship between maternal care and depressive symptoms with empathy. Related studies concerning paternal care need to be conducted by expanding the sample size. In addition, measures should be taken to obtain objective reports from participants and their parents in future studies. Finally, although our research model has a theoretical basis and empirical support, it is unable to determine causality due to its cross-sectional design. Therefore, in the future, this investigation should be extended to a longitudinal cohort study, which could reveal changes in empathy, the degree of subclinical depressive symptoms, and the degree of care received by mothers in the same group of students from the first to the fifth year to further study the relationships among empathy, subclinical depressive symptoms and maternal care among medical students of different grades.

Despite the above shortcomings, this study also has important theoretical and practical value. It is necessary to explore and develop problem-oriented educational interventions that target specific groups of medical students with measurable short- and long-term outcomes to improve the treatment of subclinical depression in medical students. This study shows the positive effects of parental care on the academic life and mental health of medical students, especially female medical students. Overall, empathy has an indirect effect, which is a noteworthy linkage mechanism. The prevalence of mental health problems among Chinese medical students is high and empathy is low. There is a lack of effective strategies to improve the situation. There is an urgent need for random intervention studies involving Chinese medical students [10, 53]. This study suggests that parental care plays an important role in direct and indirect psychological development, although the extent and mechanism of this relationship require further exploration to provide direction for future intervention studies by increasing available, low-cost, easily implemented education intervention measures to improve the mental health of medical students associated with depressive symptoms and empathy. Therefore, it is necessary to adopt measures to intervene and provide parental education, especially regarding maternal care [22]. Furthermore, each academic year, medical students are exposed to the clinical aspects of the medical profession and competence at an early age. By seeing more patients, medical students are able to have greater access to role models, which is an effective way to improve empathy [29].

## Conclusions

In summary, it is important to pay attention to medical students (especially women) with low maternal care and low empathy (cognitive empathy in particular). These students are more likely to have depressive symptoms during their medical studies and clinical work. Medical universities can integrate resources and wisdom to build a positive campus environment. Relying on professional teachers and conducting regular screening are ways to pay attention to this process. We suggest that empathy should be taught in Chinese medical schools to improve students’ interpersonal communication and transpositional consideration. Early exposure to clinical surgery training, in-depth treatment of patients and adaptation to clinical work as early as possible can enhance the empathy of medical students and reduce the possibility of depression.

## Data Availability

The datasets used and/or analysed during the current study are available from the corresponding author on reasonable request.

## Abbreviations

PBI: Parental Bonding Instrument
PBI-M: a maternal version of PBI; PBI-F: a paternal version of PBI
SDS: Self-Rating Depression Scale
JSPE-S: The Student Version of the Jefferson Empathy Scale
SAS: Self-Rating Anxiety Scale
